# Activated protein C improves intestinal microcirculation in experimental endotoxaemia in the rat

**DOI:** 10.1186/cc5093

**Published:** 2006-11-13

**Authors:** Christian Lehmann, Konrad Meissner, Andreas Knöck, Stephan Diedrich, Dragan Pavlovic, Matthias Gründling, Taras Usichenko, Michael Wendt, Jürgen Birnbaum

**Affiliations:** 1 Klinik und Poliklinik für Anästhesiologie und Intensivmedizin, Ernst Moritz Arndt University, Fr.-Loeffler-Str. 23a, D-17475 Greifswald, Germany; 2 Washington University Medical Center, Department of Anesthesiology, 660 S. Euclid Ave., St. Louis, MO 63110, USA; 3 Charité – Universitätsmedizin Berlin, Kliniken für Anästhesiologie und operative Intensivmedizin, Campus Charité Mitte, Charitéplatz 1, D-10117 Berlin, Germany

## Abstract

**Introduction:**

Successful treatment of severe sepsis and septic shock remains a major challenge in critical care medicine. The recently introduced recombinant human activated protein C (APC) remarkably improved the outcome of septic patients. The influence of APC on intestinal circulation is still poorly understood. Therefore, the present study aimed to investigate the effects of APC on intestinal microcirculation during experimental endotoxaemia in rats by using intravital microscopy.

**Methods:**

A total of 44 male Lewis rats were randomly assigned to receive intravenous injections of 15 mg/kg lipopolysaccharide alone (LPS) (*n *= 11) or LPS followed by subsequent injection of 2 mg/kg recombinant human APC (LPS + APC) (*n *= 11), whereas control animals received either APC (*n *= 11) or saline (*n *= 11). Animals underwent observations of functional capillary density and leucocyte adherence on venular endothelium in the microcirculation of the intestinal wall by means of intravital fluorescence microscopy. Indicators of macrocirculation as well as plasma levels of tumour necrosis factor-α, interleukin (IL)-1β, IL-6, and IL-10 were measured.

**Results:**

Although APC administration of both LPS-treated and control rats did not change macrocirculation or release of inflammatory cytokines, it increased mucosal and muscular functional capillary density (*p *< 0.001 and *p *< 0.05, respectively) and reduced the number of firmly adhering leucocytes in intestinal submucosal V1 and V3 venules (*p *< 0.01) in LPS + APC-treated compared with LPS-treated animals, which did not receive APC. No remarkable differences that could be attributed to APC treatment were observed between the two control groups.

**Conclusion:**

APC administration during experimental endotoxaemia improved intestinal microcirculation by protecting functional capillary density as a measure of microvascular perfusion and exerted anti-inflammatory effects by reducing leucocyte adherence to the endothelium in submucosal venules. Therefore, beneficial effects of APC in septic patients might be due, in part, to improved intestinal microcirculation.

## Introduction

Sepsis, severe sepsis, and septic shock represent progressive stages of the same illness, in which a systemic response to an infection mediated by endogenous mediators leads to a generalised inflammatory reaction in organs remote from the initial insult and eventually to organ dysfunction and/or failure [1]. Impairment of gut perfusion is regarded as one important mechanism in the development of sepsis. The splanchnic perfusion is reduced early in the course of any circulatory shock [2]. The mucosa of the gut suffers most as it experiences a high oxygen demand even in steady state [2]. Intestinal mucosal hypoperfusion with subsequent ischaemia during endotoxaemia might cause a breakdown of the gut barrier function with translocation of bacteria and their toxins into the systemic circulation, thus maintaining a 'gut-derived' septic state [3]. Gut mucosal hypoperfusion plays a major role in the pathogenesis of ongoing sepsis and multiple organ dysfunction syndrome [4] because subsequent ischaemia leads to translocation of endotoxin [5,6] and induces both vasoconstriction and hypoperfusion of small intestinal microcirculation [7]. A number of animal experiments using several different agents have aimed to improve microcirculation, particularly of the intestine, in septic conditions [4,6,8,9]. There is an increasing body of evidence that activated protein C (APC) exerts beneficial effects in the microcirculation. Human plasma-derived and human cell-produced recombinant protein C inhibits E-selectin-mediated cell adhesion to the vascular endothelium [10]. APC also attenuated endotoxin-derived pulmonary vascular injury in rats by inhibiting activated leucocytes [11]. Recently, published studies investigated the effects of APC on microcirculation during experimental endotoxaemia by intravital fluorescence microscopy (IVM) [12,13]. They were able to show that APC diminishes endotoxin-derived reduction of functional capillary density (FCD) as well as leucocyte adherence to the endothelium in dorsal skinfold chamber preparations and in the mesentery, but they did not investigate the intestinal wall. With respect to the role of the intestinal microcirculation in sepsis [14], the aim of our study was to evaluate the effects of APC administration during experimental endotoxaemia in the terminal ileum wall of the rat by using IVM.

## Materials and methods

### Animals

After approval by the local standing committee on animal experiments, a total of 44 male Lewis rats were used in the experiments (body weight 250 ± 50 g; Department of Laboratory Animal Science, Karlsburg, Ernst Moritz Arndt University, Greifswald, Germany). All experimental procedures were performed according to German animal safety legislations. Animals were kept under 12-hour light/dark rhythmic conditions (temperature 22°C, humidity 55% to 60%). Standard diet and water were available *ad libitum*. After the experiment, all animals were euthanised by overdose of intravenous (i.v.) pentobarbital.

### Anaesthesia and preparation

Anaesthesia was induced via intraperitoneal administration of 60 mg/kg pentobarbital. Maintaining of anaesthesia was achieved with repeated i.v. injections of 5 mg/kg pentobarbital (Fagron GmbH & Co. KG (previously Synopharm GmbH & Co. KG) Barsbüttel, Germany). With the animals positioned in a supine position, polyethylene catheters (PE 50, internal diameter 0.58 mm, external diameter 0.96 mm; Portex, brand of Smiths Medical, Hythe, Kent, UK) were introduced into the left external jugular vein and common carotid artery. A continuous monitoring of arterial blood pressure and heart rate (HR) was thereby undertaken (Hewlett-Packard monitor, Model 66S; Hewlett-Packard, Saronno, Italy). All animals received a tracheostomy to permit access to the airway. The animals spontaneously breathed room air. A specially tempered microscopy bench served to maintain a continuous body temperature of 37°C ± 0.5°C. Subsequent to shaving and disinfection, median laparotomy was performed from the xyphoid process to the symphysis.

### General protocol

The experiment started after a 15-minute equilibration period following preparation. Animals were randomly assigned to one of four groups (*n *= 11, respectively). In 22 animals, endotoxaemia was induced by administration of 15 mg/kg lipopolysaccharide (LPS) from *Escherichia coli*, serotype O111:B4 (Sigma-Aldrich, Steinheim, Germany). The 22 control animals were given an equivalent amount of saline. Eleven animals out of each group received 2 mg/kg APC (Drotrecogin alpha [activated], Xigris^®^; Lilly Deutschland GmbH, Bad Homburg, Germany) immediately after endotoxin or saline administration, respectively.

### Intravital fluorescence microscopy

IVM was performed 2 hours after the onset of the experiment. The examination was directed upon an isolated segment (approximately 5 cm) of the terminal ileum proximal to the ileocaecal valve, held in place by a supporting device. A coverslip served as a transparent cover. By means of this method, approximately 1 cm^2 ^of gut surface could be evaluated by microscopy. Areas of the intestine not being examined were covered with gauze and continuously superfused with isotonic saline kept at 37°C to avoid dehydration and exposure to ambient air. IVM was performed using the epifluorescent microscope Axiotech Vario (Carl Zeiss, Jena, Germany), light source HBO 50 (Carl Zeiss), oculars ×10 (Carl Zeiss), lens ×20/0.5 Achroplan (Carl Zeiss), filter type no. 20 (Carl Zeiss) for examinations with Rhodamine 6G solution (Sigma-Aldrich), filter type no. 10 (Carl Zeiss) for examinations with fluorescein isothiocyanate (FITC)-albumin, a black-and-white CCD (charge-coupled device) video camera (BC-12; AVT-Horn, Aalen, Germany), an S-VHS video tape recorder (Panasonic NV-SV120EG-S; Matsushita Audio Video GmbH, Lüneburg, Germany), and a black-and-white monitor (PM-159; Ikegami Electronics [Europe] GmbH, Neuss, Germany). Within the described configurations, a total magnification of ×500 at the 14-inch monitor was achieved. Initially, staining of the leucocytes was performed through the i.v. injection of 200 μl of 0.05% Rhodamine 6G solution. The microscope was then set to focus on the submucosa of the prepared intestinal section. Five visual fields containing non-branching, grade I stretching venules (V1) over a length of at least 300 μm, as well as another five visual fields revealing similar grade III venules (V3), were observed and recorded for 30 seconds. Two hundred microlitres of 5% FITC-albumin solution (Sigma-Aldrich) dissolved in normal saline was subsequently given to facilitate a better evaluation of the capillary flow bed through the resultant amplified contrast of the plasma. After focus setting, five video sequences (30 seconds each) of random fields of the capillaries within the longitudinal musculature as well as five fields of the capillaries within the circular muscle were recorded. Then, a section of the intestinal lumen (2 cm, antimesenteric) was opened using a microcautery knife (Geiger Model-100; Geiger Medical Technologies, Inc., Council Bluffs, IA, USA) to facilitate the examination of the mucosa. Sections filled with chymus were preferred to avoid heat damage of the opposing mesenteric wall. After flushing with isotonic saline kept at body temperature, the intestine was once again lifted and held by the supporting device. Sections of the mucosa directly bordering the mesentery were examined to circumvent possible influences from microcauterisation. Again, five video sequences (30 seconds each) of randomly chosen mucosa sections were recorded. Evaluation of all the video sequences took place off-line on a video monitor. Leucocyte adherence (the number of leucocytes that during an observation period stayed immobile for at least 30 seconds on an oblique, cylindrical endothelial surface; units, n/mm^2^) and FCD (the length of capillaries with observable erythrocyte perfusion in relation to a predetermined rectangular field; units, cm/cm^2 ^or cm^-1^) were determined according to Schmid-Schoenbein *et al*. [15].

### Laboratory analysis

Blood samples (0.55 ml) were taken at the beginning and the end of the experiments for arterial blood gas and haematocrit analysis (ABL 330; Radiometer, Hamburg, Germany). Moreover, 280 μl of plasma was fractionated and stored at -70°C for cytokine analysis (tumour necrosis factor-α [TNF-α], interleukin [IL]-1β, IL-6, and IL-10) using Rat-Quantikine ELISA [enzyme-linked immunosorbent assay] kits (R&D Systems GmbH, Wiesbaden-Nordenstadt, Germany) according to the manufacturer's instructions.

### Statistical analysis

Data analysis was performed with a statistical software package (SigmaStat; Jandel Scientific, Erkrath, Germany). All data were expressed as group means ± standard deviation and analysed using a one-way analysis of variance followed by the Newman-Keuls multiple comparison test. Mean arterial pressure (MAP) and HR were analysed by a two-way analysis of variance (repeated measures in the factor of time) followed by the Newman-Keuls multiple comparison test. A *p *value of less than 0.05 was considered significant.

## Results

### Haemodynamic changes in the macrocirculation

**Figure 1:**
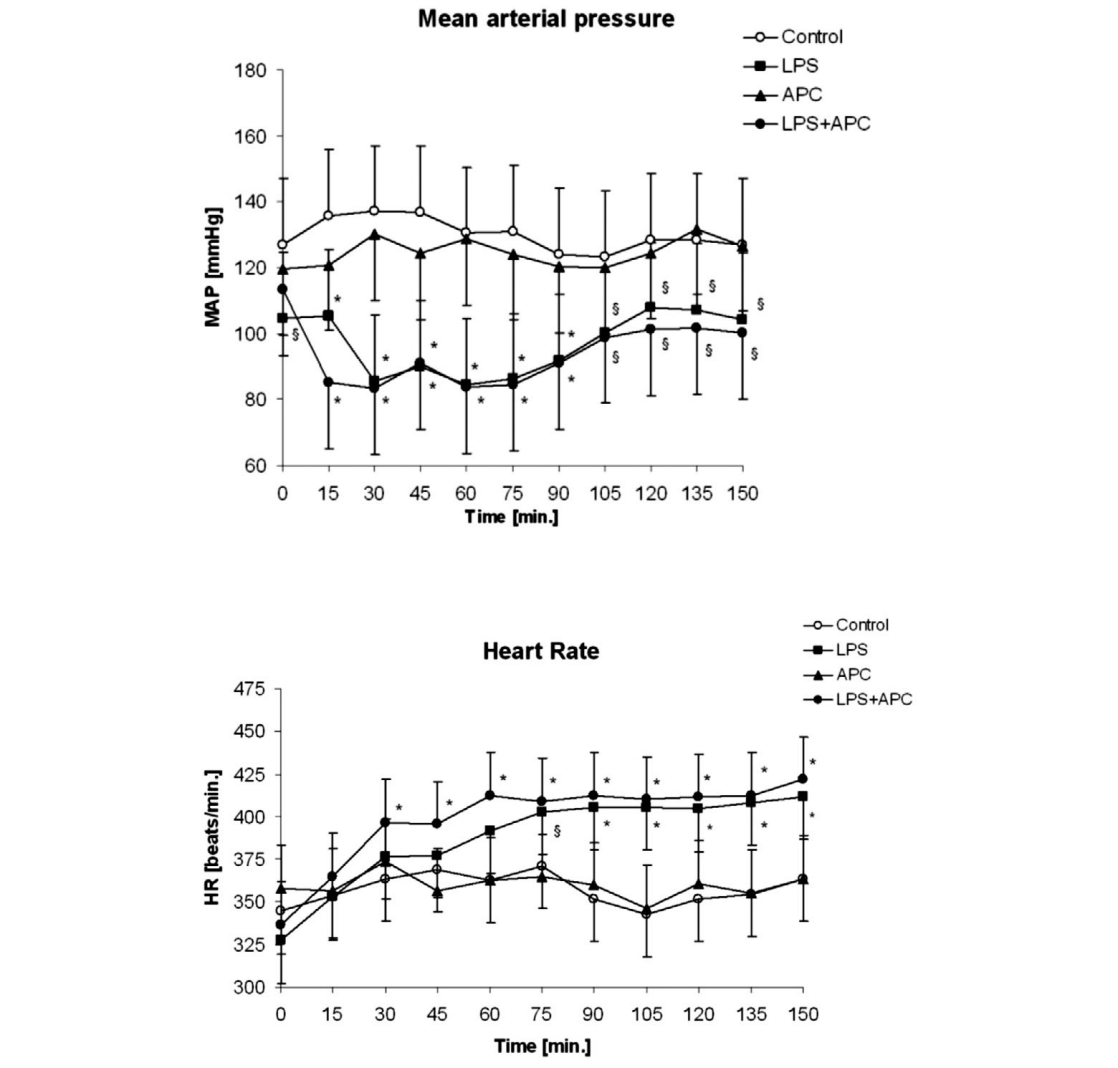
Haemodynamic data. Mean arterial pressure **(a) **and heart rate **(b)**. *p < 0.01 versus control; §*p *< 0.05 versus control. APC, activated protein C-only group; LPS, lipopolysaccharide-only group; LPS + APC, activated protein C-treated endotoxaemic group; MAP, mean arterial pressure.

MAP and HR remained stable in the non-LPS control groups (Figure 1). Endotoxin challenge resulted in a significantly decreased MAP after 30 minutes (Figure 1a). MAP was stabilised in both endotoxaemic groups two hours after LPS administration. LPS groups with and without APC treatment did not differ in MAP or HR two hours after endotoxin challenge. HR of the endotoxaemic groups was still significantly increased compared with the control groups at this time point (Figure 1b).

### Functional capillary density

**Figure 2:**
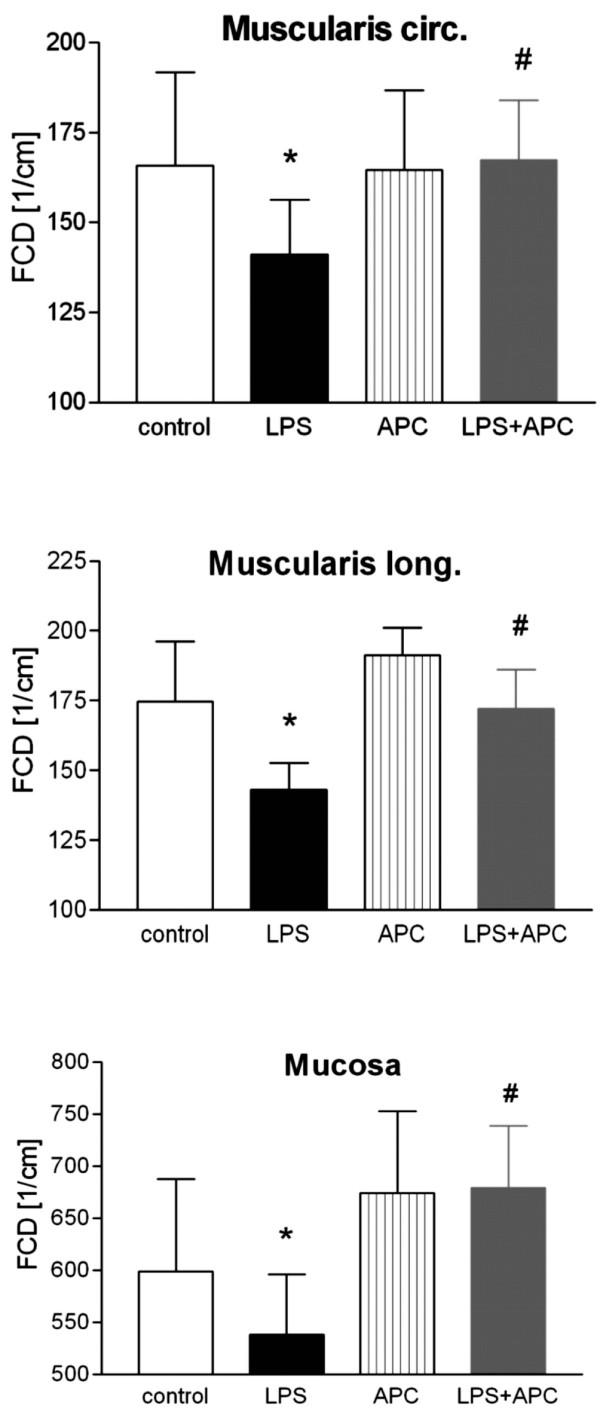
Functional capillary density (FCD) in the circular **(a) **and longitudinal **(b) **muscularis layer and in the mucosal layer **(c)**. *p < 0.05 LPS versus control; #*p *< 0.05 versus LPS. APC, activated protein C-only group; LPS, lipopolysaccharide-only group; LPS + APC, activated protein C-treated endotoxaemic group.

Changes in the FCD could be attributed to the treatment regimens of the study. Two hours after endotoxin challenge, a significant reduction of the FCD in both the circular and the longitudinal muscular layers of LPS-treated animals were observed. APC administration prevented the LPS-induced decrease of mucosal and both muscular FCDs (all *p *< 0.001; Figure 2).

### Leucocyte adherence

**Figure 3:**
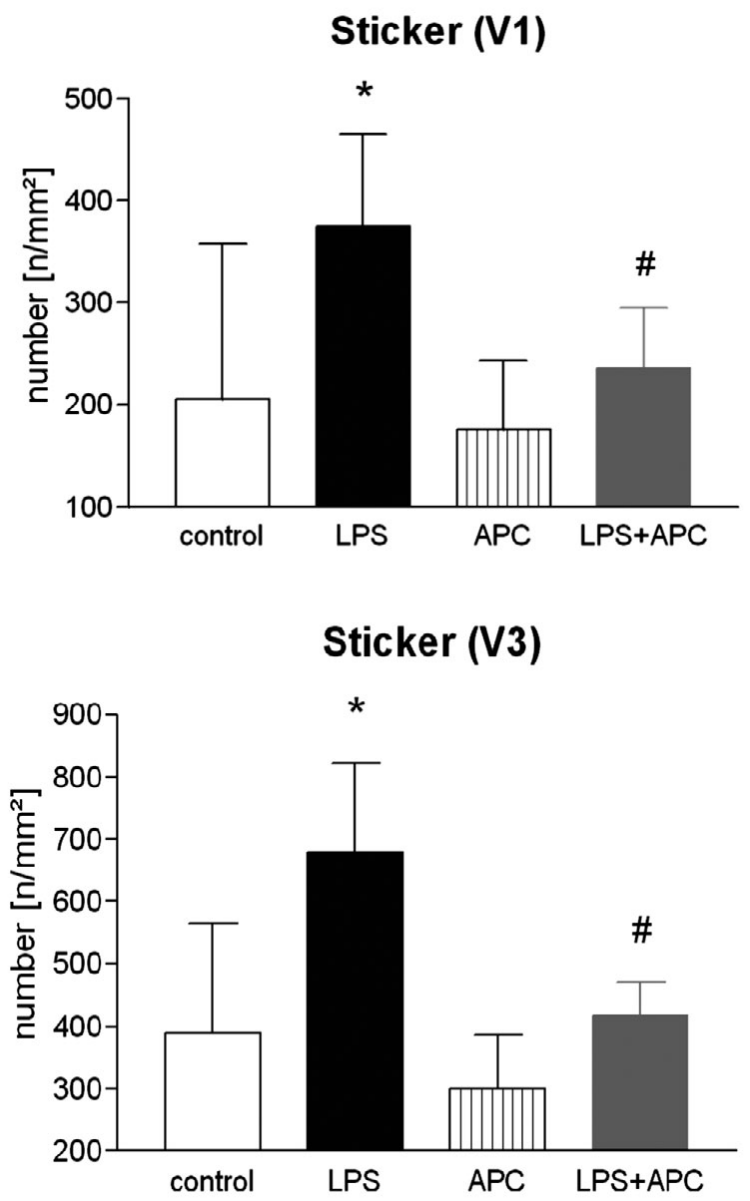
Number of closely adherent leucocytes (sticker) in V1 **(a) **and V3 **(b) **venules. *p < 0.05 versus control; #*p *< 0.05 versus LPS. APC, activated protein C-only group; LPS, lipopolysaccharide-only group; LPS + APC, activated protein C-treated endotoxaemic group.

Figure 3 shows the number of firmly adherent leucocytes two hours after endotoxin challenge. In the untreated LPS group, we saw an increase in the number of sticking leucocytes in the postcapillary venules (+45% versus control group; *p *< 0.01). In the collecting venules (V1), we saw similar effects as in the V3 venule subpopulation (+43% versus control group; *p *< 0.001). In the V3 venules of the APC-treated animals, the increase was significantly attenuated (-40% versus LPS group; *p *< 0.01). There was also an attenuation of the increase in the number of sticking leucocytes in the V1 venules (*p *< 0.01 versus LPS group).

### Blood gas and IL analysis

Blood gas and haematocrit analysis did not differ between APC-treated and control animals, which compares to the fact that we did not observe bleeding complications. LPS significantly increased inflammatory cytokines as well as IL-10 compared with the control groups (Table 1). However, APC treatment did not affect cytokine release.

## Discussion

In the present study, we showed that APC administration improved FCD as a measure of microvascular perfusion in the intestinal wall during experimental endotoxaemia. Moreover, APC treatment revealed anti-inflammatory effects by reducing leucocyte adherence to the endothelium in the intestinal submucosal venules. To the best of our knowledge, these findings have not been reported for the intestinal wall, which is one of the key sites for the manifestation of bacterial sepsis and thus for all treatment strategies for severe sepsis and septic shock alike.

There are several biologic activities of APC, besides the inhibition on coagulation, which may affect the microcirculation. Important actions of APC are also the profibrinolytic effect by inhibiting plasminogen activator-inhibitor [16] as well as anti-inflammatory actions via limited leucocyte-endothelium interaction. It could be shown that APC significantly inhibited leucocyte activation in renal ischaemia/reperfusion [17] as well as in LPS-induced pulmonary injury [11]. Two recent studies showed the beneficial effect of APC on microvascular perfusion and leucocyte-endothelium interaction in the dorsal skinfold chamber preparation of hamsters and in rat mesentery during experimental endotoxaemia [12,13].

In clinical studies, APC treatment reduced the mortality in patients with severe sepsis [18]. Furthermore, it is known that acquired protein C deficiency leads to higher mortality of septic patients [19]. Despite difficulties in the bedside diagnosis, an impaired microcirculation of various organs is frequently assumed in the clinical course of sepsis. Intestinal microcirculatory blood flow especially is diminished, and subsequent hypoxaemia impairs mucosal barrier function [2,20]. The reasons for the impairment of capillary perfusion in sepsis are manifold and not yet entirely understood [21]. One mechanism under consideration is the increased leucocyte adhesion to the endothelium, which can be visualised by IVM [12,13] and was confirmed in our work regarding intestinal microcirculation. Piper *et al*. [22] investigated leucocyte activation and flow behaviour in the microcirculation of septic rat skeletal muscle. As anticipated, leucocyte adhesion increased in the first 24 hours after sepsis. But interestingly, after 24 to 48 hours, they found a decrease of leucocyte adhesion in postcapillary venules in correlation to the reduction of circulating white blood cell count. From these data, they concluded that leucocyte adhesion is not responsible for the heterogeneity in microcirculatory blood flow. Another possible cause of a reduced blood flow in the microcirculation is the activation of coagulation. Although APC has anticoagulatory effects, other potent inhibitors of coagulation (such as antithrombin III) fail in reducing mortality of septic patients compared with APC [23]. Taking into consideration the multifactorial actions of APC on microvascular distress, which are closely linked to inflammation and coagulation [24], it becomes evident that intravital microscopy of the intestinal wall, which is described in the present study, might represent a potent tool for gaining more insight into the actions of APC.

Several cytokines have been implicated in the development of systemic inflammatory response syndrome and sepsis [25]. High levels of circulating TNF-α, IL-1β, IL-6, IL-8, and IL-10 have been shown to be linked to morbidity and mortality in septic patients. Up to now, there has been no clear evidence that APC has a direct influence on the release of inflammatory mediators. On the one hand, several animal studies have suggested anti-inflammatory effects of APC due to a reduced production of inflammatory cytokines. APC significantly inhibited the ischaemia/reperfusion-induced increase of TNF-α as well as IL-8 [17] and prevented pulmonary vascular injury by inhibiting cytokine production [26]. Iba *et al*. [13] observed a significant reduction of TNF-α and IL-6 release in rats with a much lesser endotoxin challenge (4.5 mg/kg body weight) in comparison with our model. On the other hand, these findings could not be reproduced in any clinical study yet [24]. The effects of APC could be related to the way LPS is administered.

To interpret the results of our experimental study, it is important to take into consideration the limitations of an animal model of sepsis. This setting reflects a clinical situation only contingently. To induce sepsis-like conditions, we used a single-bolus systemic LPS injection, and so development of the septic state is different from most clinical circumstances, in which a local infection is often the point of origin. Furthermore, the potentially underlying mechanisms of the effect of APC on cytokine release (for instance, inhibition of LPS-induced TNF-α production and inhibited activation of nuclear factor-κB by LPS [27,28]), on cellular activation, or on microcirculation cannot be elucidated using our setting. Also, the dosage of APC in our setting (single bolus) is different from the dosage of 24 μg/kg per hour that was used in the PROWESS (Protein C Worldwide Evaluation in Severe Sepsis) study because of the different behaviour of human APC in rats [13,29].

## Conclusion

The aim of the present study was to investigate the effects of APC on the intestinal microcirculation during experimental endotoxaemia. We found an improved FCD and a reduced leucocyte adherence in submucosal venules of the intestinal wall. Our results suggest that APC treatment could be able to slow down the 'motor' function of the intestine in sepsis with respect to the development of multiple organ failure because of its beneficial effect on the impaired intestinal microcirculation. To verify this observation in humans, clinical studies are necessary. Recently published work using orthogonal polarisation spectral imaging [30,31] as well as sidestream dark-field imaging [32] has shown that these methods could be used to monitor the microcirculation of human organs in septic state, most commonly looking at the sublingual microcirculation.

## Key messages

• APC treatment improves capillary perfusion in an endotoxin model in rats.

• APC reduced leucocyte adherence in submucosal venules of the intestinal wall as a step in the inflammation cascade.

• The anti-inflammatory properties and the beneficial effect of APC on the impaired intestinal microcirculation seem to be an important mechanism in treatment of septic patients.

## Abbreviations

APC = activated protein C; FCD = functional capillary density; FITC = fluorescein isothiocyanate; HR = heart rate; IL = interleukin; i.v. = intravenous; IVM = intravital fluorescence microscopy; LPS = lipopolysaccharide; MAP = mean arterial pressure; TNF-α = tumour necrosis factor-α; V1 = grade I venule; V3 = grade III venule.

## Competing interests

AK received a reimbursement for an oral presentation from Lilly Deutschland GmbH. Activated protein C was provided by Lilly Deutschland GmbH.

## Authors' contributions

CL and JB planned the study, established the experimental setup, and drafted the manuscript. CL conducted part of the microscopy experiments. AK conducted animal and microscopy experiments as well as data evaluation and contributed to the manuscript. SD conducted animal as well as microscopy experiments. DP contributed to the experimental setup. MG, TU, and MW contributed to the statistical evaluation of the data. KM took part in the planning and setup of the experiments, contributed to data evaluation, and wrote part of the manuscript. All authors read and approved the final manuscript.

## Figures and Tables

**Table 1:** Cytokine levels

	Control	LPS	APC	LPS + APC
TNF-α	355 ± 243	1,766 ± 168^a^	402 ± 297	1,773 ± 138^a^
IL-1β	106 ± 108	3,298 ± 1,355^a^	65 ± 21	4,820 ± 1,554^a^
IL-6	579 ± 283	8,366 ± 642^a^	604 ± 263	8,336 ± 439^a^
IL-10	246 ± 142	1,698 ± 359^a^	267 ± 150	2,076 ± 219^a^

Cytokine levels (pg/ml) two hours after endotoxin challenge (mean ± standard deviation). ^a^*p *< 0.05 versus controls. APC, activated protein C-only group; IL, interleukin; LPS, lipopolysaccharide-only group; LPS + APC, activated protein C-treated endotoxaemic group; TNF-α, tumour necrosis factor-α.
